# Novel metabolic interactions and environmental conditions mediate the boreal peatmoss-cyanobacteria mutualism

**DOI:** 10.1038/s41396-021-01136-0

**Published:** 2021-11-29

**Authors:** Alyssa A. Carrell, Dušan Veličković, Travis J. Lawrence, Benjamin P. Bowen, Katherine B. Louie, Dana L. Carper, Rosalie K. Chu, Hugh D. Mitchell, Galya Orr, Lye Meng Markillie, Sara S. Jawdy, Jane Grimwood, A. Jonathan Shaw, Jeremy Schmutz, Trent R. Northen, Christopher R. Anderton, Dale A. Pelletier, David J. Weston

**Affiliations:** 1 Biosciences Division, Oak Ridge National Laboratory, Oak Ridge, TN 37831 USA; 2 Environmental Molecular Sciences Laboratory, Pacific Northwest National Laboratory, Richland, WA 99352 USA; 3 Environmental Genomics and Systems Biology Division, Lawrence Berkeley National Laboratory, Berkeley, CA USA; 4 Department of Energy Joint Genome Institute, Lawrence Berkeley National Laboratory, Berkeley, CA USA; 5 HudsonAlpha Institute for Biotechnology, Huntsville, AL 35806 USA; 6 Department of Biology, Duke University, Durham, NC 27708 USA

**Keywords:** Molecular ecology, Plant ecology, Bacteria, Metabolomics

## Abstract

Interactions between *Sphagnum* (peat moss) and cyanobacteria play critical roles in terrestrial carbon and nitrogen cycling processes. Knowledge of the metabolites exchanged, the physiological processes involved, and the environmental conditions allowing the formation of symbiosis is important for a better understanding of the mechanisms underlying these interactions. In this study, we used a cross-feeding approach with spatially resolved metabolite profiling and metatranscriptomics to characterize the symbiosis between *Sphagnum* and *Nostoc* cyanobacteria. A pH gradient study revealed that the *Sphagnum–Nostoc* symbiosis was driven by pH, with mutualism occurring only at low pH. Metabolic cross-feeding studies along with spatially resolved matrix-assisted laser desorption/ionization mass spectrometry imaging (MALDI-MSI) identified trehalose as the main carbohydrate source released by *Sphagnum*, which were depleted by *Nostoc* along with sulfur-containing choline-O-sulfate, taurine and sulfoacetate. In exchange, *Nostoc* increased exudation of purines and amino acids. Metatranscriptome analysis indicated that *Sphagnum* host defense was downregulated when in direct contact with the *Nostoc* symbiont, but not as a result of chemical contact alone. The observations in this study elucidated environmental, metabolic, and physiological underpinnings of the widespread plant–cyanobacterial symbioses with important implications for predicting carbon and nitrogen cycling in peatland ecosystems as well as the basis of general host-microbe interactions.

## Introduction

Associations between *Sphagnum* peat mosses and phototrophic microorganisms were first reported more than a century ago, q.v. Limpricht [1], who described the presence of *Nostoc* within the dead water-filled hyaline cells of plant leaflets [2]. Subsequent work confirmed these associations in sites across the Stordalen mire of Swedish Lapland [3, 4] and the vicinity of Uppsala, Sweden. These sites encompass numerous *Sphagnum*-containing habitats, including a coniferous forest, a large mire, a nutrient-rich fen, and wetland sites exposed to human activity [5]. These early studies revealed that several algae/cyanobacterial genera are associated with the outside surfaces of *Sphagnum* as epiphytes, whereas genus *Nostoc* is most commonly found within plant cells as an endophyte. In the bryophyte hornwort, endophytic cyanobacteria receive sugars from the host plant in exchange for fixed nitrogen (N) in the form of ammonia [6–8]. However, the molecules exchanged in the *Sphagnum*–cyanobacteria symbiosis remain unknown, and it is not clear whether different molecules are exchanged during *Sphagnum* symbioses with epiphytic and endophytic cyanobacteria.

Cyanobacteria associated with *Sphagnum* fix N_2_ at higher rates than those not associated with *Sphagnum*, and in symbioses, N_2_ fixation can occur at low pH [2]. It has been hypothesized that *Sphagnum* hyaline cells have a relatively buffered pH as a consequence of cell wall cation exchange, suggesting that they serve as a “microbiome oasis” under harsh, low-pH pore water conditions [9, 10]. A field study [11] also suggested that pH influences the *Sphagnum–*cyanobacteria symbiosis. Van den Elzen et al. [11] found that N_2_ fixation activity was stimulated by the addition of bicarbonate, whereas *Sphagnum* growth remained unaffected, implying that N_2_-fixers give N to the host plant in exchange for shelter from low pH.

*Sphagnum* spp.-dominated peatlands provide a dramatic example of how changes in species interactions can cascade across levels of biological organization. These peatlands occupy just 3% of the Earth’s land surface, yet store ~25–30% of the planet’s soil carbon as dead recalcitrant peat [12]. Together with their associated N_2_-fixing bacteria, they provide a critical N input to peatland ecosystems [13, 14]. Despite the importance of this unique symbiosis to plant growth, ecosystem productivity, and even ecosystem carbon and nutrient cycling, we lack a basic understanding of the environmental conditions that allow this symbiosis to form, which metabolites are exchanged, and the physiological processes underlying these symbiotic interactions. Endophytic plant—cyanobacteria associations are best characterized and classified as nutritional symbioses where the cyanobacteria provision the plant host with fixed-N products in exchange for host derived reduced C. These mutualistic symbioses are typically facultative, and span a broad plant host phylogeny including hornworts, liverworts, angiosperms and cycads [15, 16]. Epiphytic plant—cyanobacteria symbioses are less studied, yet emerging results from the feathermoss *Pleurozium schreberi* system is providing an initial characterization. In this association, host nitrogen limitation is similarly necessary for the induction of motile cyanobacteria hormogonia production, taxis and host plant colonization [17]. However, some difference exists. For example, cyanobacteria carbon fixation and photosynthetic gene expression remains high, suggesting that the cyanobacteria may be autotrophic and not receiving reduced C from the plant host [18]. In a follow-up study, Stuart et al. [19] used cyanobacteria targeted mutagenesis coupled with stable isotope probing and high lateral resolution secondary ion mass spectrometry (NanoSIMS) to confirm mutualism as inferred from bidirectional C and N exchange, and also supported a role for organic sulfur. Such studies highlight both similarities and differences among endophytic and epiphytic plant—cyanobacteria associations and brings to question how plants that support both forms of symbiosis, like *Sphagnum* spp., physiologically operate.

The overarching aim of this study was to provide an initial characterization of the physiological and metabolic reprogramming necessary for the *Sphagnum*–*Nostoc* symbiosis and to determine what role, if any, pH plays in the establishment of symbiosis. Specifically, we tested the following hypotheses: (1) that the *Sphagnum*–*Nostoc* symbiosis is dependent on the surrounding environmental context, especially regarding pH; and (2) that the dual endophytic and epiphytic nature of the *Sphagnum*–*Nostoc* symbiosis results in unique host and bacterium physiology, metabolism, and metabolic exchange relative to other plant—cyanobacteria symbioses. To test these hypotheses, we characterized the *Sphagnum*–*Nostoc* symbiosis through metabolic cross-feeding studies, spatially resolved metabolic profiling and metatranscriptomes. The spatially resolved metabolic profiling was optimized to detect disaccharides and sulfur-rich compounds [20, 21], and allowed the placement of symbiotic partners within proximity of each other so metabolites can be sensed and exchanged in an arrangement that complemented the cross-feeding study. Furthermore, *Sphagnum* has microbially filled hyaline cells resulting in a higher microbe to plant cell ratio compared to other plant-microbe systems, which provides an ideal system for performing metatranscriptomics analysis for both host and *Nostoc* without the need of biasing RNAseq enrichment protocols.

## Materials and methods

### Stain selection and culture conditions

To identify a symbiotically competent *Nostoc* strain for our symbiosis experiments, preliminary co-culture experiments were performed to test 18 strains from the UTEX Culture Collection of Algae (University of Texas–Austin) for consistent and frequent endophytic colonization. The preliminary evaluation was conducted as described below for growth experiments at pH 5.5. *Nostoc* spp. strains UTEX 1037, UTEX1632, UTEX 2209, UTEX LB 1833, UTEX 2210, UTEX 1621, UTEX B 1545, UTEX 1038, UTEX B 384, UTEX B EE21, UTEX B EE34, UTEX B EE20, UTEX B EE7, UTEX B 2494, UTEX B EE4, UTEX B EE5, UTEX B 2493, UTEX B 2492 were evaluated for colonization within hyaline cells (i.e., endophytic colonization). Based on this preliminary study, *Nostoc muscorum* UTEX 1037 was selected as a high-colonizing strain (Fig. S1 image, Supplemental Methods). To further evaluate the suitability of *N. muscorum* UTEX 1037 as an *Sphagnum angustifolium* (Russow) C.E.O. cyanobiont, a phylogenetic tree was constructed from 37 NCBI available cyanobacterial genomes including two cyanobacteria known to colonize moss (*N. punctiforme* and *N. KVJ20* [22]) and 3 cyanobacterial genomes isolated from moss [18]. The tree used a concatenated alignment of 31 proteins with dense sampling of the *Nostoc punctiforme* species group, *Anabaena* species, and two outgroup taxa *Acaryochloris spp*. CCMEE 5410 and *A. marina* MBIC11017 (Fig. S1, Supplemental Methods). The relatively close branching of *N. muscorum* UTEX 1037 to other moss associated isolates motivated our final selection of this strain for further experimentation.

*Nostoc* strains were cultivated in 250-mL Erlenmeyer flasks in 100 mL of BG-11_0_ [23] medium (pH 8.2). The flasks were shaken at 125 rpm at 24 °C with a 16 h/8 h (day/night) cycle at 150 PAR for 21 days. *S. angustifolium* plants were collected from the SPRUCE study in Marcell Experimental Forest (MN, USA) [24]. *S. angustifolium* plants were exposed to multiple washes of 0.5–1.0% sodium hypochlorite to generate axenic plant cultures; washes were repeated until there was no visual microbial contamination which was confirmed via genome sequencing (Phytozome v13, *S. angustifolium* v1.1). Axenic *S. angustifolium* cultures were maintained on Knop’s [25] medium at pH 5.7 with a 16 h/8 h (day/night) cycle at 150 PAR.

### Sphagnum and Nostoc growth experiments

An axenic *S. angustifolium* individual (upper 2 cm portion) and/or 30 mg of (fresh weight) of *N. muscorum* UTEX 1037 were used to inoculate 2 ml of BG-11_0_ at pH 3.5, 5.5, or 8.5 in a 2-ml Eppendorf tube. Six replicates were sampled for each treatment after incubation at 24 °C with a 16 h/8 h (day/night) cycle at 150 PAR for 14 days and oven dried for 48 h. Dry weight of *S. angustifolium* grown individually was compared to the sum of the dry weight *N. muscorum* UTEX 1037 and *S. angustifolium* individually grown as well as the dry weight of *N. muscorum* UTEX 1037 *and S. angustifolium* grown together using Kruskal-Wallis with multiple testing correction using FDR (False Discovery Rate) cutoff ≤ 0.05 in R v3.6.3.

### Cross-feeding experiment

Four replicates of *N. muscorum* UTEX 1037 (1 g wet weight) and four replicates of axenic *S. angustifolium* (~1 g wet weight) were each cultured individually in 75 ml of BG-11_0_ at pH 5.5 and shaken at 125 rpm at 24 °C with a 16 h/8 h (day/night) cycle at 150 PAR for 21 days. Cultures were centrifuged at 3360 *g* for 10 min at 4 °C to pellet organisms. Pellets were equally divided, immediately frozen and stored at −80 °C for exometabolite (*n* = 4 for each organism) and RNA-seq (*n* = 4 of each organism) analyses. Supernatants were sterile-filtered through a 0.22-µm filtration device, lyophilized, resuspended in 2 mL 100% methanol, and centrifuged to pellet salts. Supernatants were equally divided for exometabolite analysis or supplementation and dried under vacuum (SpeedVac). Supernatant samples for exometabolite analysis (four replicates of each organism) were stored at −80 °C and the remaining supernatant samples (four replicates of each organism) were pooled, redissolved and supplemented to BG-11_0_ medium. For exometabolite and RNA-seq analysis, four replicates of each organism were cultured for 4 weeks in the supplemented BG-11_0_ media.

### Exometabolite analysis from cross-feeding study

Metabolites were extracted from both cell and media samples for LC-MS metabolomics analysis. Pelleted cells in 2 mL Eppendorfs were lyophilized dry (FreeZone 2.5 Plus, Labconco), then bead-beaten with a 3.2 mm stainless steel bead for 5 s (3×) in a bead-beater (Mini-Beadbeater-96, BioSpec Products) to powder. For extraction, 1 mL of 100% MeOH was added to each sample, then each briefly vortexed, sonicated in a water bath for 10 min, then centrifuged for 7 min at 7000 rpm to pellet cellular debris. Supernatant was then transferred to a 2 mL Eppendorf, dried in a SpeedVac (SPD111V, Thermo Scientific), and stored at −80 °C. Extraction controls were prepared similarly but using empty tubes exposed to the same extraction procedures.

In preparation for LC-MS/MS analysis, dried extracts were resuspended by adding 300 µL 100% methanol containing various internal standard compounds (15 µM, mix of _13_C-_15_N labeled amino acids, #767964; 1 µg/mL, 2-Amino-3-bromo-5-methylbenzoic acid, #R435902; 10 µg/mL, d4-lysine, #616192; 5 µg/mL, _13_C-_15_N-phenylalanine, #608017; 2 µg/mL, 9-anthracene carboxylic acid, #A89405; 5 µg/mL, 3,6-dihydroxy-4-methylpyridazine, #668141; 10 µg/mL, d5-benzoic acid, #217158, 10 µg/mL, 4-(3,3-dimethyl-ureido)benzoic acid, #CDS014672—Sigma) vortexed briefly, sonicated in a water bath for 10 min, then centrifuged (5 min at 5000 rpm). 150 µL of resuspended extract was centrifuge-filtered (2.5 min at 2500 rpm) through a 0.22 µm filter (UFC40GV0S, Millipore), then transferred to a glass autosampler vial.

Samples were analyzed via LC-MS on a system consisting of an Agilent 1290 UHPLC coupled to a Thermo QExactive Orbitrap HF (Thermo Scientific, San Jose, CA) mass spectrometer. Normal phase chromatography was performed by injecting 2 µL extract into a zic-HILIC column (Millipore SeQuant ZIC-HILIC, 150 ×2.1 mm, 5 µm, Cat# 50454) warmed to 40 °C. The column was equilibrated with 100% buffer B (95:5 ACN:H2O w/ 5 mM ammonium acetate) for 1.5 min at 0.45 mL/min, diluting buffer B down to 65% with buffer A (H2O w/ 5 mM ammonium acetate) for 13.5 min, down to 0% B over 3 min while increasing flow to 0.6 mL/min, and followed by isocratic elution in 100% buffer A for 5 min. MS and MS/MS data were collected in both positive and negative ion mode using, with full MS spectra acquired ranging from 70 to 1050 *m/z* at 60,000 resolution, and fragmentation data acquired using an average of stepped collision energies of 10, 20, and 30 eV at 17,500 resolution. Orbitrap instrument parameters included a sheath gas flow rate of 50 (au), auxiliary gas flow rate of 20 (au), sweep gas flow rate of 2 (au), 3 kV spray voltage and 400 °C capillary temperature. Sample injection order was randomized, and an injection blank of methanol only run between each sample.

Metabolites were identified based on exact mass and retention time coupled with comparing MS/MS fragmentation spectra to compound standards. LC-MS data was analyzed using custom Python code [26]. Features (unique *m/z* coupled with retention time, RT) were assigned a score from 0 to 3, representing the level of confidence in compound identification and then rated according to the Metabolomics Standards Initiative (MSI) confidence levels [27]. A positive level 1 identification was given for compounds detected at *m/z* < / = 5 ppm or 0.001 Da from theoretical as well as RT < / = 0.5 min compared to a pure standard run using the same LC-MS method. A compound with the highest level of positive identification (score of 3), exceeding level 1, also had matching MS/MS fragmentation spectra in comparison to either an outside database (e.g., METLIN) or internal database generated from standards run and collected on a Q Exactive Orbitrap HF MS. Mismatches in MS/MS invalidated as an identification. Starting total ion count of metabolites in the cross-fed supernatant were compared to the ending supernatant metabolite total ion count using in R v3.6.3. Starting total ion count of metabolites in the cross-fed supernatant were compared to the ending supernatant metabolite total ion count using Kruskal-Wallis with multiple testing correction using FDR cutoff ≤ 0.05 in R v3.6.3.

### MALDI-MSI analysis

Samples were prepared for matrix-assisted laser desorption/ionization mass spectrometry imaging (MALDI-MSI) analysis using a modification of a workflow we described previously [21]. Briefly *S. angustifolium* and *N. muscorum* UTEX 1037 were positioned 2 cm apart for co-culture or individually in the center (controls) of BG-11_0_ (pH 4.5) 1.5% agar plates and incubated at 24 °C with a 16 h/8 h (day/night) cycle until the interaction zones were analyzed. Agar areas of the *N. muscorum* UTEX 1037—*S. angustifolium* interaction and of isolated culture controls were excised from Petri dishes and placed onto double-sided adhesive copper tape adhered to indium tin oxide-coated glass slides (Bruker Daltonics). The copper tape approach enhanced our sensitivity for analysis in negative ionization mode and improved adhesion of agar onto the MALDI target. Notably, the moss protruded from the surface of the agar, so plant tissue was carefully removed prior to dehydration to assist in MS analyses. Samples were dried at 40 °C overnight, after which MALDI matrix was applied using a HTX TM-Sprayer (HTX Technologies). For analysis in positive-ion mode, universal MALDI matrix (1:1 2-5-dihydroxybenzoic acid: α-Cyano-4-hydroxycinnamic acid), 20 mg/mL in 70% MeOH, was applied using the following spraying conditions: 12 passes with a track spacing of 3 mm, flow rate of 0.1 mL/min, spray velocity 1,200 mm/min, spray pressure of 10 psi (N_2_), and a 40-mm distance from the sprayer nozzle to the sample. For analysis in negative-ion mode, 7 mg/mL of N-(1-naphthyl) ethylenediamine dihydrochloride (NEDC) in 70% MeOH was sprayed with eight passes at 1,200 µL/min, 75 °C, a spray spacing of 3 mm, and a spray velocity of 1200 mm/min. MALDI-MSI was performed on a 15-Tesla Fourier transform ion cyclotron resonance (FTICR)-MS (Bruker Daltonics) equipped with SmartBeam II laser source (355 nm) using 200 shots/pixel with a frequency of 2 kHz and a step size of 200 µm. FTICR-MS was operated to collect m/z 92–700, using a 209-ms transient, which translated to a mass resolution of *R* ~ 120,000 at 400 m/z. Data were acquired using FlexImaging (v 4.1, Bruker Daltonics), and image processing and visualization were performed using SCiLS (Bruker Daltonics).

### RNA sequencing and analysis

RNA was extracted from pelleted *N. muscorum* UTEX 1037 or *S. angustifolium* grown in BG-11_0_ and BG-11_0_ supplemented with exometabolites from the corresponding organism (*n* = 4 for each treatment) by a combined method using CTAB lysis buffer and the Spectrum Total Plant RNA extraction kit (Sigma). Approximately 100 mg of flash-frozen ground tissue was incubated in 850 µl of CTAB buffer (1.0 % β-Me) at 56 °C for 5 min; 600 µl chloroform:isoamylalcohol (24:1) was added, and the samples were centrifuged for 8 min. The supernatant (~730 µl) was transferred to a filter column provided in the Spectrum kit, and the standard Spectrum kit protocol was followed accompanied by DNase treatment. Ribo-zero bacterial (Illumina cat#MRZB12424) and plant (Illumina cat#MRZSR116) were used to enrich the transcripts. The Applied Biosystems SOLiD Total RNA-Seq kit (catalog number 4445374) was used to generate the cDNA template library. Briefly, the mRNA was fragmented by chemical hydrolysis followed by ligation with strand-specific adapters and reverse transcription to generate cDNA. cDNA fragments of 150–250 bp were isolated, and the SOLiD EZ Bead system was used to perform emulsion clonal bead amplification to generate bead templates for SOLiD platform sequencing. Samples were sequenced on the 5500XL SOLiD platform. The 50-base short-read sequences produced by the 5500XL SOLiD sequencer were mapped in color space against the genomes of *Sphagnum angustifolium v1.1* (Phytozome v13, phytozome-next.jgi.doe.gov/) and *N. muscorum* UTEX 1037 draft genome with the SOLiD LifeScope software version 2.5, using default parameters.

Reads from *N. muscorum* UTEX 1037 were assessed for quality using htseq-qa [28]. Sequence regions showing GC content instability were trimmed using an R script. Reads were assembled using SPAdes version: 3.10.1 [29] using -k 21,33,55,77,99,127. Best assembly quality (as assessed by QUAST [30]) was acquired using 25% of the reads. Gene annotation was performed with Prokka [31], and gene sets for photosynthesis, carbon fixation, and nitrogen fixation were identified using HMMs with HMMER [32]. Plant gene models were assigned to MapMan4 ontology for gene set enrichment. Gene set enrichment analysis (GSEA) of differentially expressed genes in *S. angustifolium* was performed using MapMan4 gene ontologies on the desktop version of MapMan. MapMan4 gene ontologies were assigned using the Mercator 4 v2.0 web portal [33]. To determine significance, Kruskal–Wallis tests within the MapMan desktop version, with multiple testing correction using FDR cutoff ≤ 0.05, were performed in R v3.6.3. GSEA was performed similarly for *N. muscorum* UTEX 1037.

## Results

### Growth study

#### The Sphagnum–Nostoc symbiosis is dependent on pH

**Fig. 1:**
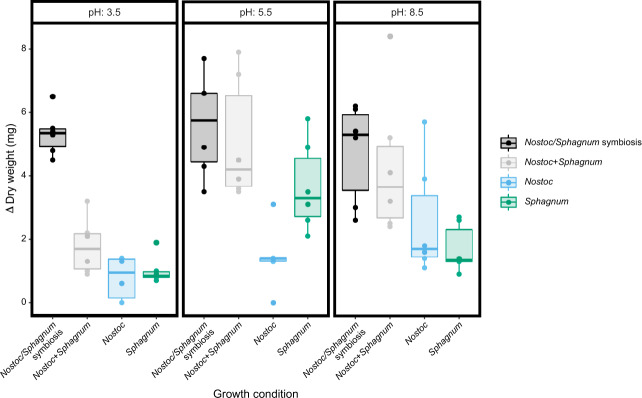
*Sphagnum angustifolium* (*Sphagnum*) and *Nostoc muscorum* UTEX 1037 (*Nostoc*) dry weight growth analysis across variable pH conditions. At pH 3.5, growth was highest when both organisms were grown together (Table S1). Growth of *Sphagnum* grown individually or with *Nostoc* across a pH gradient were determined by the final dry weight (mg) of organisms grown in BG-11_0_ (*n* = 6). Samples labeled with “symbiosis” represent the two organisms grown together, and *Nostoc* + *Sphagnum* shows the sum of individual growth of each organism alone.

To test the hypothesis that variable pH conditions influence *S. angustifolium*—*N. muscorum* UTEX 1037 symbiosis, we grew germ-free *S. angustifolium angustifolium* and *N. muscorum* UTEX 1037 at pH 3.5, 5.5, and 8.5. Symbiosis outcome was evaluated by comparing the growth of *S. angustifolium* in direct contact with *N. muscorum* UTEX 1037 (designated *Nostoc/Sphagnum symbiosis*), relative to individual growth in isolation and the sum of individual growth (designated *Nostoc* + *Sphagnum*). There was a clear benefit to symbiosis in the low pH (3.5) environment: when the species were grown together under these conditions, the change in weight was greater than the sum of individual growth (Fig. 1; FDR adjusted *p* = 0.012). However, the growth benefit was not clearly observed at pH of 5.5 or 8.5 (Table S1).

#### Metabolic cross-feeding study identifies the utilization and release of 225 exometabolites

**Fig. 2:**
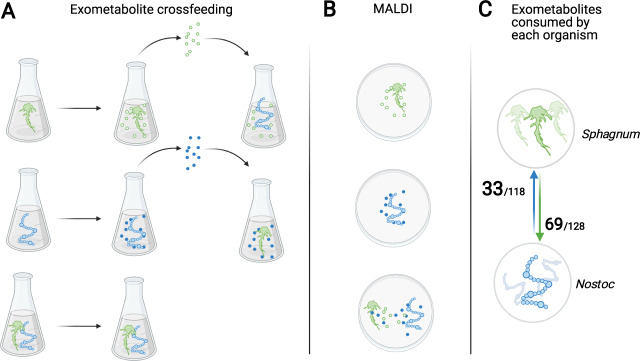
Experimental design and approach. **A** For the cross-feeding study, each organism was cultured in BG-11_0_ followed by characterization by liquid chromatography-mass spectrometry (LC-MS). The spent medium was then cross-fed in a full factorial design in which the resultant spent medium and cell extracts were profiled using LC-MS. **B** Experimental design of MALDI-MSI analysis of metabolites produced by each organism alone or when grown together in close proximity. **C** The number of statistically significant identified cross-fed exometabolites depleted by each organism out of all exometabolites exuded by the symbiosis partner.

The growth benefits observed for both *N. muscorum* UTEX 1037 and *S. angustifolium* demonstrate a mutualistic symbiosis at low pH and motivated us to characterize the metabolic exchange mediating this interaction. Therefore, we sought to assess the metabolites being released by each partner of the symbiosis when grown in fresh liquid BG-11_0_ medium and the change in abundance of the released exometabolites in spent medium when cross-fed to the other partner (Fig. 2A). Overall, we identified 225 exometabolites from the spent medium of individual partners when cultured in fresh BG-11_0_ medium (Table S2). Of the identified exometabolites, *N. muscorum* UTEX 1037 53% of exometabolites exuded by *S. angustifolium* and *S. angustifolium* depleted 28% of exometabolites exuded by *N. muscorum* UTEX 1037 (Fig. 2C).

**Fig. 3:**
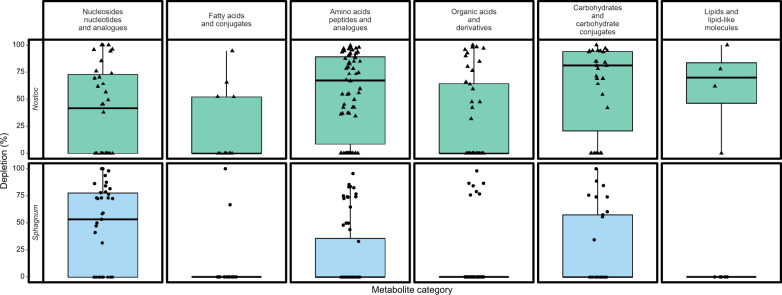
Distribution of *Sphagnum angustifolium* (*Sphagnum*) and *Nostoc muscorum* UTEX 1037 (*Nostoc*) depletion of partner produced metabolites presented as percent depleted. Rows show metabolites depleted by each partner, columns indicate the class of the metabolite, and colors indicate the source of the metabolite (green = *Sphagnum*, blue=*Nostoc*). Boxes span from the 25th to the 75th percentile. The horizontal line indicates the median, the error bars are the range of data or 1.5 interquartile range, and the points represent individual metabolites; *n* = 4.

To further characterize metabolite exchange, the exometabolites that significantly (*p* < 0.05) changed in abundance before and after feeding were categorized into six chemical classes: amino acids, carbohydrates, fatty acids and their conjugates, lipids, nucleotides and nucleosides, and organic acids (Fig. 3). *N. muscorum* UTEX 1037 depleted large percentages from each chemical class of *S. angustifolium* exometabolites: 75% of the measured amino acids, 77% of the carbohydrates, 50% of the fatty acids, 68% of the lipids, 35% of the nucleotides/nucleosides, and 62% of the organic acids. Within each class, log-fold change (LFC) analysis revealed that the exometabolites that changed the most after cross-feeding were as follows: the pyrimidine nucleotide sugars uridine 5’-diphosphogalactose (−14.53 LFC, *p* value = 0.01) and uridine 5′-diphosphoglucose (−14.5 LFC, *p* value = 0.01); the amino acids arginine (−7.87 LFC, *p* value = 0.02), acetyl-L-alanine (−5.59 LFC, *p* value = 0.02), and asparagine (−5.45 LFC, *p* value = 0.02); the organic acid sulfuric acid ester choline-O-sulfate (−5.62 LFC, *p* value = 0.02); and the carbohydrate trehalose (−2.7 LFC, *p* value = 0.02). Thus, these compounds represent *S. angustifolium* exometabolites depleted by *N. muscorum* UTEX 1037 (Table S3).

When fed *N. muscorum* UTEX 1037 exometabolites, *S. angustifolium* depleted 30% of the identified amino acids, 54% of the carbohydrates, and 76% of the nucleosides. Among the depleted metabolites within each class, LFC analysis revealed that the most depleted exometabolites were as follows: the nucleosides cytidine (−17.7 LFC, *p value* = 0.01), xanthosine (−5.67 LFC, *p value* = 0.02), adenine (−3.98 LFC, *p value* = 0.02), 2’-deoxyguanosine (−2.43 LFC, *p value* = 0.02), and uridine (−2.66 LCF, *p value* = 0.02); the amino acids amino acid derivatives aspartate (−4.49 LFC, *p value* = 0.02), acetyl-methionine (−2.77 LFC, *p value* = 0.02), arginine (−2.6 LFC, *p value* = 0.02), glutamic acid (−1.8 LFC, *p value* = 0.02); and the carbohydrates and carbohydrate derivatives phosphoglyceric acid (−3.12 LFC, *p value* = 0.02) and gluconate (−2.6 LFC, *p value* = 0.02) (Table S4).

#### Spatial metabolite profiling corroborates the exchange of trehalose and xanthosine in the Sphagnum–Nostoc symbiosis

We further investigated the results of the cross-feeding study using ultrahigh mass resolution and mass accuracy MALDI-MSI. This approach allowed us to place the symbiotic partners within proximity of each other, allowing metabolites to be sensed and exchanged in an arrangement that complemented the cross-feeding study. We hypothesized that metabolites critical for symbiosis would increase in abundance when the symbiosis partner was present.

**Fig. 4:**
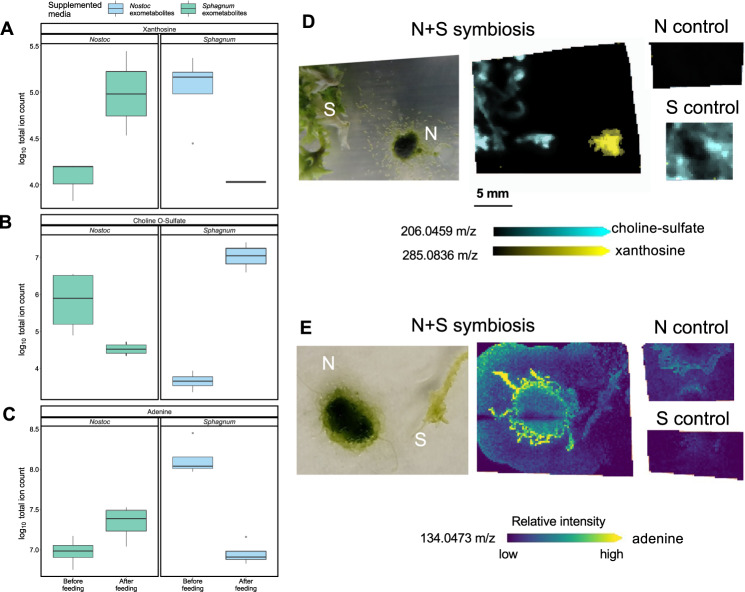
Cross-feeding and MALDI-MSI results for choline-O-sulfate, xanthosine, and adenine. Boxplots of exometabolite cross-feeding of *Sphagnum angustifolium* (*Sphagnum*) and *Nostoc muscorum* UTEX 1037 (*Nostoc*) spent media demonstrate an exchange of xanthosine (**A**), choline O-sulfate (**B**), and adenine (**C**) between *Nostoc* and *Sphagnum*. Boxes span from the 25th to the 75th percentile *n* = 4. The horizontal line indicates the median, the lines denote the max and min values, outliers are displayed as dots. Exchange of choline O-sulfate by *Sphagnum* for *x*anthosine provided by *Nostoc* (**D**) and an increase in production of adenine by *Nostoc* in the presence of *Sphagnum* (**E**) were also confirmed by MALDI-MSI.

**Fig. 5:**
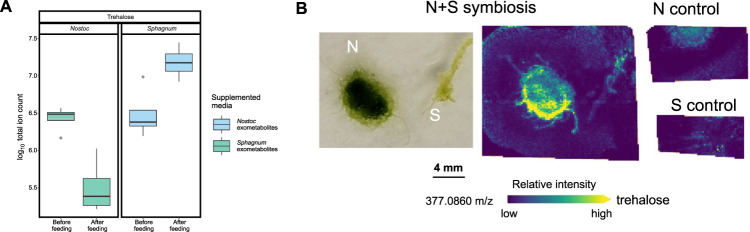
Cross-feeding and MALDI-MSI results for trehalose exchange. Boxplots of exometabolite cross-feeding of *Sphagnum angustifolium* (*Sphagnum*) and *Nostoc muscorum* UTEX 1037 (*Nostoc*) spent media demonstrate significant exudation of trehalose by moss fed cyanobacteria exometabolites and depletion of trehalose by *Nostoc* when fed *Sphagnum* exometabolites (**A**). Boxes span from the 25th to the 75th percentile *n* = 4. The horizontal line indicates the median, the lines denote the max and min values, outliers are displayed as dots. Negligible exudation of trehalose was detected by MALDI-MSI in moss alone, but increased exudation was detected when *Nostoc* (N) and *Sphagnum* (S) were grown together (**B**).

To test this hypothesis, we first targeted candidate metabolites identified from the cross-feeding study and investigated their spatially resolved abundance profiles across the interaction zone. Using this approach, we found that *N. muscorum* UTEX 1037 and *S. angustifolium* produced small amounts of xanthosine when grown individually; however, when the species were grown in proximity to each other, *N. muscorum* UTEX 1037 increased xanthosine production, whereas *S. angustifolium* did not (Fig. 4D). Furthermore, a cross-feeding experiment showed that *S. angustifolium* depleted *N. muscorum* UTEX 1037 provided xanthosine (from ave. ion count 1.4 × 10^5^, sd 0.8 × 10^5^ to ave. ion count 0.02 × 10^5^, sd 0.05 × 10^5^), whereas *N. muscorum* UTEX 1037 did not deplete the *S. angustifolium* -provided xanthosine (from ave. ion count 1.0 × 10^7^, sd 0.37 × 10^7^ to ave. ion count 1.0 × 10^7^, sd 0.4 × 10^7^) (Fig. 4A). Similar results were observed for adenine (Fig. 4E): cross-feeding revealed that *S. angustifolium* depleted adenine released from *N. muscorum* UTEX 1037 from ave. ion count 14.9 × 10^7^, sd 8.9 × 10^7^ to ave. ion count 0.9 × 10^7^, sd 0.3 × 10^7^), and *N. muscorum* UTEX 1037 released elevated levels of adenine when supplemented with *S. angustifolium* exudates from ave. ion count 1.0 × 10^7^, sd 0.38 × 10^7^ to ave. ion count 2.4 × 10^7^, sd 1.0 × 10^7^) (Fig. 4C). Trehalose distribution was investigated similarly. Cross-feeding analysis revealed a clear trend in production of putative trehalose by *S. angustifolium*, with *N. muscorum* UTEX 1037 consuming up to 85% of the exometabolite (Fig. 5A). The abundance of putative trehalose being produced by *S. angustifolium* was similar between individual *S. angustifolium* cultures in fresh BG-11_0_ medium (ave. ion count 2.8 × 10^6^, sd .94 × 10^6^) or after being fed *N. muscorum* UTEX 1037 exometabolites (ave. ion count 16.0 × 10^6^, sd 8.3 × 10^6^). The MALDI-MSI data support these findings: ion abundance was similar in isolated *S. angustifolium* and *S. angustifolium* grown in proximity of *N. muscorum* UTEX 1037. However, we observed a notable increase in the abundance of the disaccharide pool in *N. muscorum* UTEX 1037 when co-cultured with *S. angustifolium*. (Fig. 5B).

#### Spatial metabolite profiling corroborates choline-O-sulfate exchange and identifies additional sulfur-rich taurine and sulfoacetate as exometabolites contributing to the symbiosis

Choline-O-sulfate was also identified in our cross-feeding study and visualized by MALDI-MSI. Ion images indicated that *S. angustifolium* produces choline-O-sulfate regardless of the presence of the *N. muscorum* UTEX 1037 partner (Fig. 4D) and that the metabolite was not detected in *N. muscorum* UTEX 1037. Cross-feeding results revealed that on average, 98% of choline-O-sulfate provided by *S. angustifolium* was depleted by *N. muscorum* UTEX 1037 (Fig. 4B). In addition, MALDI-MSI analysis showed that *S. angustifolium* produced sulfoacetate, which was excreted at high levels in the agar medium only when *N. muscorum* UTEX 1037 and *S. angustifolium* were grown together. Furthermore, MALDI-MSI revealed evidence that taurine was metabolized to sulfoacetaldehyde via 2-oxyglutarate and glutamate, and that the sulfoacetaldehyde was further converted to acetylphosphate, likely feeding into pyruvate metabolism (Fig. S2) within *N. muscorum* UTEX 1037. The induction of those metabolites, with the exception of acetylphosphate, was only observed when *N. muscorum* UTEX 1037 was cultured in proximity to *S. angustifolium*.

#### Transcriptional responses of Nostoc and Sphagnum provide insight into symbiotic interactions

Metatranscriptomes were collected at the conclusion of our cross-feeding experiment. Samples were collected from *S. angustifolium* and *Nostoc* grown separately, *S. angustifolium* and *Nostoc* cross-fed with exometabolites from the other partner, and *S. angustifolium* and *Nostoc* grown together in direct contact (Fig. 2A). Given a priori knowledge of other plant–cyanobacterium symbiosis, we hypothesized that the *S. angustifolium*—*N. muscorum* UTEX 1037 symbiosis would result in the downregulation of *N. muscorum* UTEX 1037 photosynthesis, as carbohydrates are supplied by the plant host in exchange for N-rich metabolites from the *N. muscorum* UTEX 1037 partner via enhanced N_2_ fixation [34].

**Fig. 6:**
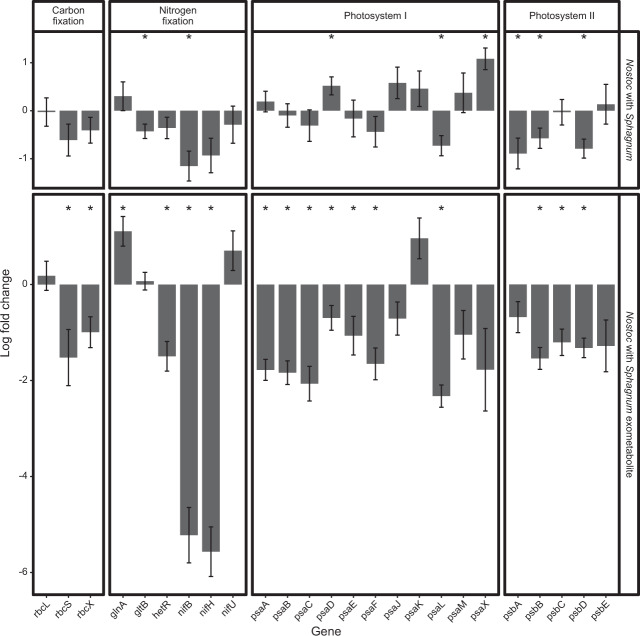
Comparative gene expression profiling of *Nostoc muscorum* UTEX 1037 (*Nostoc*) grown in direct contact with *Sphagnum angustifolium* (*Sphagnum*), or with *Sphagnum* produced exometabolites. Average log_2_ fold-change of genes related to photosystems I & II, carbon fixation, and nitrogen fixation related in *Nostoc* grown with *Sphagnum* vs. *Nostoc* grown alone. Asterisks indicate FDR-corrected *p value* ≤ 0.05. Errors bars represent ± 1 standard deviation; *n* = 4.

The results from the *N. muscorum* UTEX 1037 RNA-seq analysis targeting the N_2_ fixation pathway did not support this hypothesis (Fig. 6). Instead, genes involved in N_2_ fixation exhibited a decreasing trend, with the exception of glutamine synthetase (glnA; Fig. 6). In accordance with expectations, expression of *N. muscorum* UTEX 1037 genes involved in photosynthesis generally decreased for both photosystem I and II when the cyanobacteria were supplemented with *S. angustifolium* exometabolites, although expression of photosystem I genes tended in increase when *N. muscorum* UTEX 1037 was grown directly with *S. angustifolium*.

**Fig. 7:**
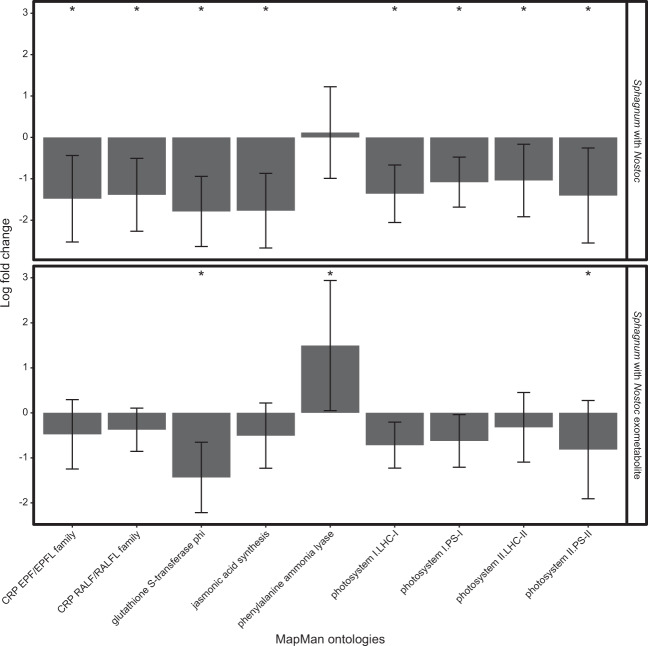
Comparative gene expression profiling of *Sphagnum angustifolium* (*Sphagnum*), grown in direct contact with *Nostoc muscorum* UTEX 1037 (*Nostoc*), or with *Nostoc* produced exometabolites. Average log_2_ fold-change of MapMan *Sphagnum* ontologies, asterisks indicate FDR-corrected *p value* ≤ 0.05 signifying significant enrichment of the MapMan ontology in differentially expressed genes. Errors bars represent ±1 standard deviation; *n* = 4.

Likewise, *S. angustifolium* RNA-seq analysis with MapMan4 ontology enrichment did not support the hypothesis that photosynthesis genes would be increased during symbiosis. However, enrichment analysis identified downregulation of genes involved in *S. angustifolium* host defense when the host was in direct contact with the *N. muscorum* UTEX 1037 (Fig. 7). For example, genes associated with cysteine rich peptides (CRP) (−1.48 LFC, *p* = 0.006), glutathione S-transferase activities (GST) (−1.4 LFC, *p* = 0.00006), and jasmonic acid synthesis (JAS) (−1.7 LFC, *p* = 0.03) were expressed at lower levels in *S. angustifolium* grown in direct contact with *N. muscorum* UTEX 1037 (Table S5) than in *S. angustifolium* fed *N. muscorum* UTEX 1037 exometabolites (Table S6). Furthermore, the plant defense–related gene phenylalanine ammonia lyase (PAL) was induced (1.49 LFC, *p* = 0.00004) when *S. angustifolium* was fed *N. muscorum* UTEX 1037 exometabolites, but the expression of the gene did not change when the organisms were in in direct contact (Fig. 7).

## Discussion

The overarching aim of this study was to provide an initial characterization of the physiological and metabolic reprogramming necessary for the *Sphagnum*–*Nostoc* symbiosis while testing the following hypotheses: 1) the *Sphagnum*–*Nostoc* symbiosis is dependent on the surrounding environmental context, particularly in regard to pH, and, 2) the dual endophytic and epiphytic nature of the *Sphagnum*–*Nostoc* symbiosis [22] results in unique host and bacterial physiology, metabolism, and metabolic exchange relative to other plant—cyanobacteria symbioses. The first hypothesis was based on field observations showing that raising the pH by addition of bicarbonate benefited the N_2_-fixing partners of the symbiosis over the *Sphagnum* host [11], leading the authors of that study to suggest that the cyanobacteria trade growth for protection under specific environmental conditions. The second hypothesis stems from the unique *Sphagnum*-*Nostoc* association that can take place epiphytically on leaflet and stem surfaces similar to the feathermoss-cyanobacteria symbiosis [18] or endophytically within *Sphagnum* host hyaline cells analogous to the enclosed cavities found in some liverwort and hornworts or the intracellular *Gunnera* system (reviewed in [16, 35]). Below, we address each hypothesis within the context of the current results and the broader plant–cyanobacteria literature.

### The Sphagnum–Nostoc symbiosis is dependent on pH

In support of our first hypothesis, we found that pH was a critical factor in determining both the formation of the symbiosis and its outcome. We observed mutualism, in which both the *S. angustifolium* host and *N. muscorum* UTEX 1037 symbiont increased their growth, only at a low pH (3.5). As pH increased to 5.5 and to 8.5, *N. muscorum* UTEX 1037 growth continued to increase growth, whereas *S. angustifolium* growth declined steeply at high pH. This finding is supported by studies that have investigated *S. angustifolium* and cyanobacteria individually. For example, previous reports on *Sphagnum* growth to variable pH levels and other environmental factors [36] revealed that growth increased from a low pH of 3.5 to a peak around 5.5, followed by a sharp decline at pH 7.5. Indeed, high pH is associated with *Sphagnum* die-off [11, 37]; this is in direct contrast to our observation of cyanobacteria, for which the same high pH conditions were associated with peak growth. This trend is consistent with field observations showing that a pH of 8.1 was associated with the highest percentage of heterocystous cyanobacteria [38]. Taken together, these findings support the notion proposed by van den Elzen et al. [11] that the N_2_-fixing diazotrophic community and *Sphagnum* host have different niche preferences, and that at low pH and sub-optimal acidic conditions the diazotrophs trade growth in exchange for protection from predation [39].

### The Sphagnum—Nostoc metabolic exchange includes amino acids, purine metabolism, and trehalose

Plant–cyanobacteria interactions are commonly characterized as nutrient exchange symbioses in which carbohydrates are produced by the plant in exchange for N-rich compounds produced by the cyanobacterial symbiont (e.g., [1, 2]). This general statement encompasses significant variation, and uncertainty remains regarding the identities of the molecules being exchanged and how this compares to other plant–cyanobacteria symbioses ranging from ephemeral epiphytic interactions (e.g., *Pleurozium* spp. [18]) to endophytic interactions such as those of *Gunnera*. Therefore, we sought to identify the metabolites being released by each partner of the symbiosis when grown in fresh liquid BG-11_0_ medium, as well as the change in abundance of those exometabolites, using both a static cross-feeding approach and a MALDI-MSI approach in which symbiotic partners were able to interact (Fig. 2A).

**Fig. 8:**
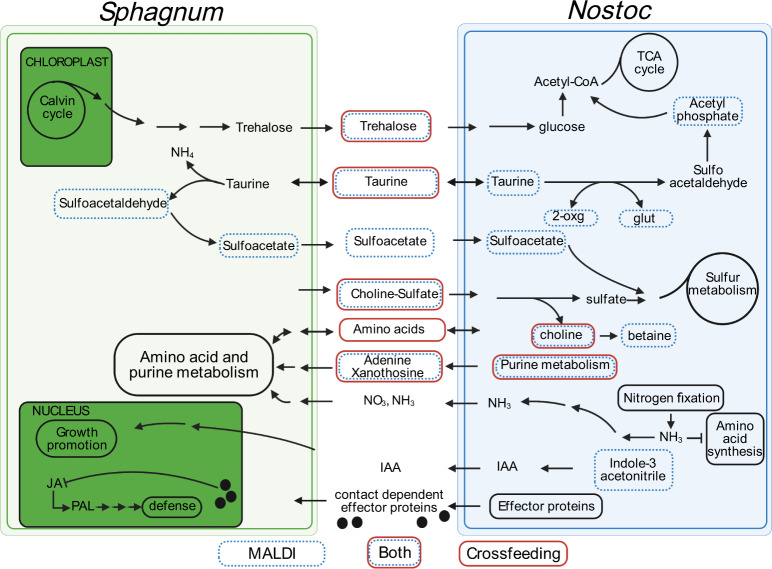
Conceptual model of *Sphagnum*–*Nostoc* symbiosis based on MALDI-MSI, cross-feeding, and metatranscriptomic analyses. Blue boxes indicate the metabolite was confirmed with MALDI-MSI, red boxes indicate the metabolite was identified in the cross-feeding experiment, and red/blue boxes represent metabolites confirmed with both MALDI-MSI and cross-feeding. Black boxes represent generalized metabolic pathways that were not evidenced by MALDI-MSI or from the cross-feeding study but have some support via metatranscriptomics (Tables S5, S6). For simplicity, metabolic processes specific to *Nostoc* heterocyst (i.e., N_2_ fixation) and vegetative cells are not separated.

*Nostoc* in symbiosis tends to decrease conversion of fixed N into amino acids through down-regulation of *glnA* (glutamine synthetase, GS) and *gltB* (glutamate synthase, GOGAT). This allows the release of fixed N as ammonium, 40–95% of which is then taken up by the plant host (reviewed in [40]). Cycad associations are an exception to this, as amino acids seem to be the main N currency [41], and the lack of change in *glnA* and *gltB* transcript levels in the epiphytic feathermoss–*Nostoc* association led Warshan et al. [18] to hypothesize that amino acids are also the main N currency for that system. In the current study, *N. muscorum* UTEX 1037 *gltB* expression was reduced only when the cyanobacteria were cultured in direct contact with *S. angustifolium*, but not when they were fed *S. angustifolium* exometabolites, suggesting that *N. muscorum* UTEX 1037 derived ammonium may act as a currency that can be exchanged only during direct symbiosis (Fig. 8). However, we must be circumspect regarding this possibility: the ammonium ion was too small for detection in the design of the current study, and our cross-feeding analyses clearly identified the transfer of the amino acids arginine, alanine, and asparagine from *N. muscorum* UTEX 1037 to the moss host, suggesting that the metabolic currency comprises multiple forms of N. Future this symbiosis supports both endophytic and epiphytic associations, thus studies should test the hypothesis that direct endophytic colonization favors inorganic ammonium as the main N currency, whereas epiphytic associations favor organic forms such as amino acids.

Cross-feeding and MALDI-MSI analysis also suggest multiple roles for purine metabolism within the *S. angustifolium—N. muscorum* UTEX 1037 symbiosis (Fig. 8). First, *N. muscorum* UTEX 1037 significantly increased release of adenine when cross-fed *S. angustifolium* exometabolites, whereas *S. angustifolium* depleted adenine when fed *N. muscorum* UTEX 1037 exometabolites. Purines, and in particular adenine, play key roles in the interactions between several nodule-forming rhizobacteria and their hosts. For example, the *Sinorhizobium fredii* purine metabolism mutant *purL*^-^ or strains with reduced *purL* expression levels fail to produce viable nodules on soybean (*Glycine max*) [42, 43]. Nodulation is also inhibited on the *Aeschynomene* host in *Bradyrhizobium purL* mutants and mutants in other purine synthesis genes [44]. Although the mechanism is not entirely understood, it is interesting to note that modifications of purine biosynthesis also inhibit symbiosis with non-plant hosts, including the *Burkholderia*–stinkbug [45] and *Photorhabdus–*nematode [46] associations, suggesting a role for purines that extend well beyond moss–*Nostoc* interactions.

We also observed a role for purine metabolism within the *S. angustifolium - N. muscorum* UTEX 1037 symbiosis involving xanthosine (Fig. 8). Similar to adenine, the abundance of exuded xanthosine increased when *N. muscorum* UTEX 1037 was cross-fed *S. angustifolium* exometabolites, whereas *S. angustifolium* depleted xanthosine when fed *N. muscorum* UTEX 1037 exometabolites. Xanthosine is a catabolite of purine nucleotides and a key constituent of RNA. It also plays a well-known role in soybean–rhizobium symbiosis in which xanthosine forms the purine base xanthine, leading to the formation of uric acid via xanthine dehydrogenase. Uricase then converts uric acid to allantoin (reviewed in [47]), which along with allantoic acid is the major transport form of N through plant host xylem [48]. By contrast, in non–N_2_-fixing soybean plants, amino acids constitute the major form of N [49]. Furthermore, recent studies extended the role of allantoin to abiotic stress tolerance through the involvement of abscisic acid and jasmonic acid [50]. The role of purine metabolism in the *S. angustifolium* – *N. muscorum* UTEX 1037 symbiosis, and more specifically allantoin as a form of N currency and its involvement in the stress response, deserves further investigation.

In general during plant–cyanobacteria symbiosis, cyanobacterial photosynthesis is downregulated, and the reduced C-fixing capacity is compensated by carbohydrates supplied by the plant. The carbohydrates exchanged in plant–cyanobacteria symbioses is usually sucrose [8], but bryophytes tend to use trehalose as a major form of carbohydrate. Indeed, our earlier work [20] in conjunction with the findings reported here clearly identify a role for *S. angustifolium* produced trehalose as the main C currency during symbiosis (Fig. 8). Consistent with this, MALDI-MSI revealed that trehalose was the only disaccharide released from *S. angustifolium*. Trehalose is a non-reducing carbohydrate, it is relatively chemically inert and very stable at low pH; moreover, it has long been recognized as an important signaling molecule in vascular plant symbiosis. Numerous studies have documented its role as a cellular osmoprotectant [51–54] leading to stress tolerance [55, 56] and even enhanced N_2_ fixation [57]. In our experiments, the abundance of *S. angustifolium*-released trehalose increased in the presence of *N. muscorum* UTEX 1037. The exudation of trehalose may simply be the consequence of a metabolic byproduct, as trehalose is a major carbohydrate form in mosses. However, the stability of trehalose within low-pH environments, along with its contribution to stress tolerance at harsh high-latitude ecosystems, may have driven co-evolutionary events that made trehalose the major form of carbohydrate in the metabolic currency.

### A possible role for sulfur metabolism in the Sphagnum–Nostoc symbiosis

Sulfur was recently suggested to play a role in the cyanobacteria–feather moss symbiosis [18]. To explore this notion in the *S. angustifolium—N. muscorum* UTEX 1037 system, we mined MALDI-MSI spectra for S-rich compounds. Choline-O-sulfate (Fig. 4D) and taurine (Fig. S2) were produced in large abundance by the moss host and depleted by *N. muscorum* UTEX 1037 (Fig. 8), e.g., 98% of choline-O-sulfate provided by *S. angustifolium* was depleted by *N. muscorum* UTEX 1037. Although choline-O-sulfate itself can serve as a compatible solute for osmotic stress regulation in bacteria and plants [58–60], some bacteria are able to metabolize this compound to yield sulfate for use as a sulfur source and choline that can further be transformed to glycine betaine, which can in turn be further metabolized to ammonia and pyruvate. Hence, it is not surprising that choline-like moieties are present in lipid head groups of bacteria occupying *Sphagnum*-dominated northern peatlands, suggesting that these compounds contribute to membrane stability under acidic conditions [17, 19, 61]. Similarly, taurine is considered a major C source for marine prokaryotes [62] and along with other organosulfur molecules is considered to be a key exchange molecule in a cosmopolitan marine diatom–bacteria symbiosis [63]. Such findings led Warshan et al. [18] to suggest that cyanobacteria in epiphytic associations do not obligately rely on the moss host for fixed C, but rather receive C as a reward from aliphatic sulfonate compounds such as taurine, as revealed in this current study. Cyanobacteria require considerable amounts of S to support the Fe–S clusters necessary for mature nitrogenase synthesis [16, 18]. Therefore, the provision of choline-O-sulfate and taurine by the moss host may benefit *N. muscorum* UTEX 1037 in multiple ways, including access to limiting nutrients for N_2_ fixation and amino acid synthesis, osmotic protection from low-pH bog conditions, and even by providing a C source.

### Host defense response to cyanobacteria colonization

Our metatranscriptome analysis revealed that *S. angustifolium* host defense was downregulated when the plant was in direct contact with the *N. muscorum* UTEX 1037 symbiont, but not as a result of chemical contact alone (Fig. 7). This conclusion was supported by reductions in expression of genes associated with cysteine rich peptides, glutathione S-transferases, and jasmonic acid biosynthesis. Furthermore, phenylalanine ammonia lyase (PAL) was induced when *S. angustifolium* was fed *N. muscorum* UTEX 1037 exometabolites, but not when *S. angustifolium* was in direct contact with *N. muscorum* UTEX 1037. Multiple studies have shown that *PAL* expression responds to environmental stressors including pathogen infection, wounding, nutrient depletion and extreme temperature change [64, 65]. In *Arabidopsis*, the quadruple mutant *pal1*/*pal2*/*pal3*/*pal4* is extremely susceptible to pathogenic bacteria, lower levels of salicylic acid, and reduced levels of lignin relative to wild-type plants [66]. Given the role of PAL in plant defense, it is not surprising that this enzyme would also play a role in symbiotic outcomes, as beneficial microbes must bypass host defense systems. In *Lotus japonicus*, for example, *PAL* expression is significantly induced 2 days after inoculation with *Mesorhizobium loti* inoculation, but dramatically suppressed after prolonged inoculation [67]. A comprehensive study by Chen et al. [68], demonstrated that PAL influences multiple processes in the *Lotus japonicus*–*Rhizobium* symbiosis, including lignin modification and salicylic acid signaling and biosynthesis. A note of caution must be placed on the current results as altered N status, and other abiotic factors could influence gene expression. Nonetheless, *N. muscorum* UTEX 1037 bypasses the *S. angustifolium* host defense system as evidenced by colonization, and how this happens remains to be determined.

## Conclusion

These observations expand our knowledge of the environmental, metabolic, and physiological underpinnings of the *S. angustifolium*—*N. muscorum* UTEX 1037 mutualism. Our findings show that mutualism is dependent on environmental context, and in particular is driven by acidic pH. Moreover, we showed that trehalose is the main disaccharide provided by the *S. angustifolium host* in exchange for N-rich nucleosides and amino acids, and their derivatives, including cytidine, xanthosine, adenine, 2’-deoxyguanosine, uridine aspartate, acetyl-methionine, arginine, glutamic acid. Although this suggests that the N provided by *N. muscorum* UTEX 1037 is organic rather than ammonium, as is often the case in plant—cyanobacteria associations, we cannot draw a strong conclusion on this issue because neither the cross-feeding or MALDI-MSI approaches we used in this study were capable of identifying ammonium. Notably in this regard, however, our metatranscriptome analysis revealed downregulation of *N. muscorum* UTEX 1037 GS-GOGAT, which is associated with the release of fixed N as ammonium. This effect was observed only when the organisms were in direct contact and not cross-fed. Thus, the environmental context of the symbiosis (i.e., pH) may not only drive the type of symbiosis, from commensal epiphytic symbiosis to mutualistic endophytic symbiosis, but may also influence the form of N currency that is exchanged. In addition to identifying C- and N-rich exometabolites, we also clearly demonstrated exchange of exometabolites for host-provided S-rich molecules such as taurine, choline-O-sulfate, and sulfoacetate. How sulfur metabolism is involved in the C and N nutrient exchange remains to be fully elucidated. It is reasonable to hypothesize that cyanobacteria need S to support the synthesis of Fe-S clusters for nitrogenase, and also benefit from the protective effects of choline-O-sulfate in harsh, low-pH peat bogs. Our conceptual model (Fig. 8) details these newly described metabolic interactions along with key hypothesized interactions the testing of which will improve our understanding of plant–cyanobacterial interactions. We note that *Sphagnum* hyaline cells can be occupied by a number of interacting micro-organisms [10, 69, 70] and that the current conceptual model will need to include additional members to ultimately enhance predictions of C and N cycling in peatland ecosystems.

## Supplementary information

Supplemental figures

Supplemental Methods

Table S1

Table S2

Table S3

Table S4

Table S5

Table S6
