# Supplementary material for: Novel metabolic interactions and environmental conditions mediate the boreal peatmoss-cyanobacteria mutualism
Source: ISME J. 2021 Nov 29;16(4):1074–85. doi: 10.1038/s41396-021-01136-0 (PMC8941135; doi:10.1038/s41396-021-01136-0)
Supplement: Supplementary file 1 — Supplemental figures [file 41396_2021_1136_MOESM1_ESM.docx]

**Supplemental Figures**


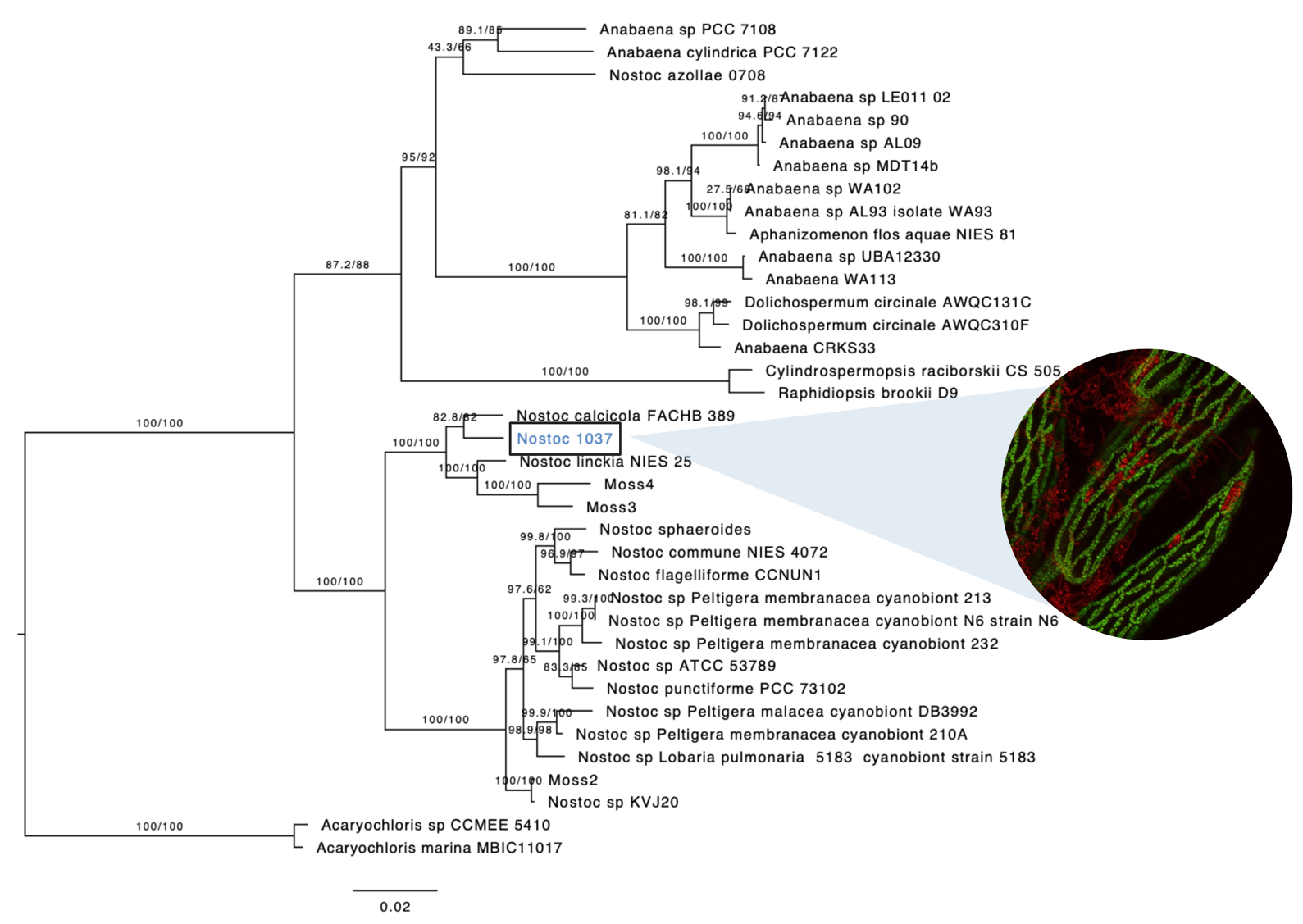


Figure S1: *Nostoc 1037,* the isolate used in this study, colonizes *Sphagnum angustifolium* and is closely related to previously identified moss-associated cyanobacteria. In this maximum-likelihood phylogram of *Nostoc 1037* and select cyanobacteria, the numbers at nodes indicate UFBoot2 and SH-like approximate likelihood ratio support. Branch lengths indicate estimated substitutions per site. Blue label indicates *Nostoc 1037*, and cyanobacteria with the name Moss2, Moss3, and Moss4 were isolated from feathermoss by Warshan et al. [14] The epifluorescence microscope image confirms *Nostoc 1037* endophytic and epiphytic colonization of *Sphagnum angustifolum.*

Figure S2: MALDI imaging of taurine and the pyruvate metabolism pathway in the interaction zone of *Sphagnum* (S) and *Nostoc* (N) relative to individually grown controls.
