# Supplementary material for: Novel metabolic interactions and environmental conditions mediate the boreal peatmoss-cyanobacteria mutualism
Source: ISME J. 2021 Nov 29;16(4):1074–85. doi: 10.1038/s41396-021-01136-0 (PMC8941135; doi:10.1038/s41396-021-01136-0)
Supplement: Supplementary file 2 — Supplemental Methods [file 41396_2021_1136_MOESM2_ESM.docx]

**Supplemental Methods**

To determine the phylogenetic relationship between *Nostoc 1037* and cyanobacteria known to associate with moss we downloaded 37 cyanobacterial genomes from NCBI representing the currently sequenced diversity of clade A (*Oscillatoria*/*Arthrospira*), clade B (*Nostoc*/*Anabaena*/*Cyanothece*) according to the nomenclature of [1] with dense sampling of *Nostoc* and *Anabaena* species, as well as two outgroup taxa *Acaryochloris* sp. CCMEE 5410 and *A. marina* MBIC11017. In addition, we included six cyanobacterial isolates sequenced in [2] *Nostoc moss2, moss3,* and *moss4.*. A concatenated alignment of 31 proteins (*frr, pyrG, pgk, tsf, rpsE, rplD, rplK, infC, rplC, rplL, rplF, rplP, dnaG, rplA, rpsK, rplM, rplS, rpsJ, nusA, rpsI, rpsM, rpsS, rpsC, rplB, rplT, rplE, rpsB, rplN, smpB, rpmA, rpoB*) for the 125 cyanobacterial genomes was generated and trimmed using the AMPHORA2 pipeline [3] with HMMER v3.2.1 [4]. Alignment sites containing only gaps and ambiguous characters were removed using FAST v1.6 [5]. Molecular evolution model selection was performed with ModelFinder [6]. We compared LG [7] and cpREV [8] empirical matrix models in combination with or without empirical profile mixture models C10-C60 [9], a proportion of invariant sites (+I), rate heterogeneity across sites using either a discrete gamma distribution with four rate categories (+4G) or the FreeRate model (+R) [10, 11] with up to 32 categories, and empirical estimated amino acid frequencies (+F) using the Bayesian Information Criterion. Phylogenetic analysis was conducted with IQ-TREE multicore version 1.6.8 [12] using the cpREV+C60+F+R6 model. Node support was evaluated using 1000 replicates of the SH-like approximate likelihood ratio test (SH-aLRT) [13] replicates and 1000 UFboot2 replicates [14, 15] with a single round of nearest neighbor interchange per UFboot2 replicate.

1. Shih PM, Wu D, Latifi A, Axen SD, Fewer DP, Talla E, et al. Improving the coverage of the cyanobacterial phylum using diversity-driven genome sequencing. *Proc Natl Acad Sci U S A* 2013; **110**: 1053–1058.

2. Warshan D, Espinoza JL, Stuart RK, Richter RA, Kim S-Y, Shapiro N, et al. Feathermoss and epiphytic *Nostoc* cooperate differently: expanding the spectrum of plant–cyanobacteria symbiosis. *ISME J* 2017; 1–13.

3. Wu M, Scott AJ. Phylogenomic analysis of bacterial and archaeal sequences with AMPHORA2. *Bioinformatics* 2012; **28**: 1033–1034.

4. Finn RD, Clements J, Eddy SR. HMMER web server: Interactive sequence similarity searching. *Nucleic Acids Res* 2011; **39**: W29–W37.

5. Lawrence TJ, Kauffman KT, Amrine KCH, Carper DL, Lee RS, Becich PJ, et al. FAST: FAST analysis of sequences toolbox. *Front Genet* 2015; **6**.

6. Kalyaanamoorthy S, Minh BQ, Wong TKF, von Haeseler A, Jermiin LS. ModelFinder: fast model selection for accurate phylogenetic estimates. *Nat Methods* 2017; **14**: 587–589.

7. Le SQ, Gascuel O. An Improved General Amino Acid Replacement Matrix. *Mol Biol Evol* 2008; **25**: 1307–1320.

8. Adachi J, Waddell PJ, Martin W, Hasegawa M. Plastid Genome Phylogeny and a Model of Amino Acid Substitution for Proteins Encoded by Chloroplast DNA. *J Mol Evol* 2000; **50**: 348–358.

9. Si Quang L, Gascuel O, Lartillot N. Empirical profile mixture models for phylogenetic reconstruction. *Bioinformatics* 2008; **24**: 2317–2323.

10. Yang Z. A space-time process model for the evolution of DNA sequences. *Genetics* 1995; **139**: 993 LP – 1005.

11. Soubrier J, Steel M, Lee MSY, Der Sarkissian C, Guindon S, Ho SYW, et al. The Influence of Rate Heterogeneity among Sites on the Time Dependence of Molecular Rates. *Mol Biol Evol* 2012; **29**: 3345–3358.

12. Nguyen LT, Schmidt HA, Von Haeseler A, Minh BQ. IQ-TREE: A fast and effective stochastic algorithm for estimating maximum-likelihood phylogenies. *Mol Biol Evol* 2015; **32**: 268–274.

13. Guindon S, Dufayard J-F, Lefort V, Anisimova M, Hordijk W, Gascuel O. New Algorithms and Methods to Estimate Maximum-Likelihood Phylogenies: Assessing the Performance of PhyML 3.0. *Syst Biol* 2010; **59**: 307–321.

14. Minh BQ, Nguyen MAT, von Haeseler A. Ultrafast Approximation for Phylogenetic Bootstrap. *Mol Biol Evol* 2013; **30**: 1188–1195.

15. Hoang DT, Chernomor O, von Haeseler A, Minh BQ, Vinh LS. UFBoot2: Improving the Ultrafast Bootstrap Approximation. *Mol Biol Evol* 2018; **35**: 518–522.
